# Dysfunctional Brain Networking among Autonomic Regulatory Structures in Temporal Lobe Epilepsy Patients at High Risk of Sudden Unexpected Death in Epilepsy

**DOI:** 10.3389/fneur.2017.00544

**Published:** 2017-10-16

**Authors:** Luke A. Allen, Ronald M. Harper, Rajesh Kumar, Maxime Guye, Jennifer A Ogren, Samden D. Lhatoo, Louis Lemieux, Catherine A. Scott, Sjoerd B. Vos, Sandhya Rani, Beate Diehl

**Affiliations:** ^1^Institute of Neurology, University College London, London, United Kingdom; ^2^Epilepsy Society, Chalfont St. Peter, United Kingdom; ^3^The Center for SUDEP Research, National Institute of Neurological Disorders and Stroke, Bethesda, MD, United States; ^4^Department of Neurobiology, David Geffen School of Medicine at UCLA, Los Angeles, CA, United States; ^5^UCLA Brain Research Institute, Los Angeles, CA, United States; ^6^Department of Anaesthesiology, David Geffen School of Medicine at UCLA, Los Angeles, CA, United States; ^7^Department of Radiological Sciences, David Geffen School of Medicine at UCLA, Los Angeles, CA, United States; ^8^Department of Bioengineering, David Geffen School of Medicine at UCLA, Los Angeles, CA, United States; ^9^Aix Marseille University, CNRS, CRMBM UMR 7339, Marseille, France; ^10^Epilepsy Centre, Neurological Institute, University Hospitals Case Medical Centre, Cleveland, OH, United States; ^11^Translational Imaging Group, University College London, London, United Kingdom

**Keywords:** graph theory, resting state, functional connectivity, hippocampus, insula

## Abstract

**Background:**

Sudden unexpected death in epilepsy (SUDEP) is common among young people with epilepsy. Individuals who are at high risk of SUDEP exhibit regional brain structural and functional connectivity (FC) alterations compared with low-risk patients. However, less is known about network-based FC differences among critical cortical and subcortical autonomic regulatory brain structures in temporal lobe epilepsy (TLE) patients at high risk of SUDEP.

**Methods:**

32 TLE patients were risk-stratified according to the following clinical criteria: age of epilepsy onset, duration of epilepsy, frequency of generalized tonic–clonic seizures, and presence of nocturnal seizures, resulting in 14 high-risk and 18 low-risk cases. Resting-state functional magnetic resonance imaging (rs-fMRI) signal time courses were extracted from 11 bilateral cortical and subcortical brain regions involved in autonomic and other regulatory processes. After computing all pairwise correlations, FC matrices were analyzed using the network-based statistic. FC strength among the 11 brain regions was compared between the high- and low-risk patients. Increases and decreases in FC were sought, using high-risk > low-risk and low-risk > high-risk contrasts (with covariates age, gender, lateralization of epilepsy, and presence of hippocampal sclerosis).

**Results:**

High-risk TLE patients showed a subnetwork with significantly reduced FC (*t* = 2.5, *p* = 0.029) involving the thalamus, brain stem, anterior cingulate, putamen and amygdala, and a second subnetwork with significantly elevated FC (*t* = 2.1, *p* = 0.031), which extended to medial/orbital frontal cortex, insula, hippocampus, amygdala, subcallosal cortex, brain stem, thalamus, caudate, and putamen.

**Conclusion:**

TLE patients at high risk of SUDEP showed widespread FC differences between key autonomic regulatory brain regions compared to those at low risk. The altered FC revealed here may help to shed light on the functional correlates of autonomic disturbances in epilepsy and mechanisms involved in SUDEP. Furthermore, these findings represent possible objective biomarkers which could help to identify high-risk patients and enhance SUDEP risk stratification *via* the use of non-invasive neuroimaging, which would require validation in larger cohorts, with extension to patients with other epilepsies and subjects who succumb to SUDEP.

## Introduction

Sudden unexpected death in epilepsy (SUDEP) is the most common cause of premature death among people with epilepsy (1), for whom the risk of sudden death is over 20 times that of the general population (2, 3). Patients at higher risk of SUDEP are individuals who experience ongoing and frequent generalized tonic–clonic seizures [GTCS (4)]. Although the underlying mechanisms remain elusive, seizure-induced autonomic (cardiac arrhythmia or hypotension) or respiratory (hypoventilation, apnea or apneusis) dysfunction, or a fatal combination of these have been postulated as likely causes (5–7). Other precipitating processes, including metabolic, hormonal, and genetic actions may contribute to SUDEP (8). However, clear pathophysiological mechanisms linking epilepsy with SUDEP remain lacking.

A recent evaluation of cardiorespiratory arrests in epilepsy monitoring units—the MORTEMUS project (9)—showed severe respiratory and cardiac alterations (apnea/hypoventilation and bradycardia/asystole) to occur terminally in cases of SUDEP. However, whether the fatal respiratory or cardiac observations in SUDEP cases are secondary to profound inhibition of central respiratory or autonomic regulatory sites (10) is unclear. All but one of the SUDEP cases reviewed in the MORTEMUS project were preceded by a GTCS and experiencing frequent GTCS (>3 per year) has been identified as a major risk factor for SUDEP (11, 12). Other key factors related to increased SUDEP risk include the occurrence of nocturnal seizures, a longer disease duration, and an earlier age of disease onset (11, 13).

Recent efforts to identify structural neuroimaging biomarkers of SUDEP have revealed volume differences within key autonomic regulatory brain structures. Voxel-based morphometry (VBM) procedures show significantly reduced bilateral posterior thalamic (pulvinar) gray matter (GM) volumes and increased right hippocampal and amygdala GM volumes in high- vs. low-risk SUDEP subjects (14). A similar volumetric approach found severe volume loss in the dorsal mesencephalon among SUDEP cases compared to epilepsy and healthy controls (15). Resting-state functional magnetic resonance imaging (rs-fMRI), a technique used to identify at-rest functional connectivity (FC) between brain areas (16), shows reduced connectivity between several key regions, including the pons, thalamus, and anterior cingulate in high- vs. low-risk SUDEP patients (17). However, it is not known how the FC involving other forebrain, limbic, and basal ganglia regions, sites that regulate autonomic and respiratory functions, is affected in patients at high risk of SUDEP. For example, FC investigations involving the hippocampus and medial and orbital frontal cortices are lacking despite their known role in blood pressure regulation (18). Since SUDEP likely involves processes incorporating failure or dysfunction of respiratory or autonomic regulation, an objective of this study was to focus on FC between areas related to autonomic and respiratory processes.

Central regulation of both autonomic and respiratory control is represented through multiple structures at several levels of the neuraxis, and extends far beyond the usually designated areas in the medulla and pons. The final pathways for respiratory as well as sympathetic and parasympathetic control are mediated through medullary areas, but multiple cortical, diencephalic, midbrain, and especially cerebellar structures contribute to activation, inhibition, and timing of both respiratory and autonomic control. Cerebellar structures include the deep fastigial nuclei, important for influences on breathing, while the cerebral cortex includes the bilateral insulae, the ventral medial prefrontal gyri, and the cingulate cortex. Subcortical structures such as the hippocampus, hypothalamus, amygdala, thalamus, and basal ganglia (particularly the caudate and putamen) are also heavily involved. These structures have repeatedly been shown to respond to autonomic or respiratory challenges and structural changes in breathing and cardiovascular conditions (19–21), send projections between each other, and many project directly to nuclei regulating respiratory and autonomic action in the brain stem (22–25).

Of particular concern is that epileptic seizures arising in, or rapidly propagating to, central autonomic control sites within the limbic system (26) result in damage to or dysregulation of critical autonomic and other regulatory structures (27). The majority of seizures are accompanied by symptoms of autonomic nervous system activation and, in some cases, dysfunction (28). Cardiac alterations, particularly increased heart rate, are found in almost all seizures (29), with some suggesting more often so in temporal lobe epilepsy (TLE) patients (30). Interictal heart rate variability (HRV) reflects autonomic imbalances in patients with poorly controlled epilepsy (31) and among those who experience GTCS (32) and reduced HRV has shown to correlate with increased SUDEP risk (33). The most severe autonomic and respiratory alterations are observed during and after GTCS (9). Ongoing GTCS could exert a profound impact on critical brain areas, potentially disrupting vital processes by which respiratory, cardiac, and blood pressure functions are regulated (34).

Little is known about how, or to what extent, brain regions involved in autonomic and respiratory regulation are affected in TLE patients as a consequence of increased SUDEP risk (which includes a higher frequency of GTCS). The main objective of the current study was to investigate potential differences in FC among a subnetwork of key structures related to autonomic and respiratory regulation. We investigated this subnetwork using rs-fMRI, and applying the network based statistic [NBS (35)] to compare FC between high and low risk of SUDEP patients. The NBS is a graph theory-based approach to FC analysis which exploits the clustering structure of between-group differences in network topology. That is, connections within a network which significantly differ across groups often form a connected subnetwork or “component.” Similar to conventional neuroimaging analysis (36), whereby clusters are identified among voxels in physical space, the NBS identifies clusters in topological space and possesses greater power to detect strength-based differences as opposed to methods which ignore such a topological structure. We hypothesized that TLE patients at high risk of SUDEP would exhibit altered FC among the subnetwork of selected autonomic regions of interest (ROIs) compared to patients at low risk of SUDEP.

## Materials and Methods

### Subjects and Risk Stratification

Sixty patients with TLE underwent rs-fMRI scanning (34 left TLE; 26 right TLE). Of these subjects, 28 were excluded from further analyses due to the presence of large lesions (9), interictal epileptic discharges (IEDs) recorded during the rs-fMRI scan acquisition (8), excessive head movement [9: 4 max head-motion (exceeding 2 mm), 5 scrubbing; see fMRI preprocessing], and two cases who suffered SUDEP. We excluded patients who suffered from SUDEP, so as not to mix potential pathological differences which may be present in these cases.

Of 32 patients remaining for further analysis, 17 had left TLE (9 females) and 15 right TLE (7 females). Subjects were classified as being at high or low risk of SUDEP based on clinical factors (11, 14, 17) as follows: An odds ratio (OR) score was generated for each patient using duration of epilepsy > 15 years (OR = 1.95), epilepsy onset < 16 years (OR = 1.72), >3 GTCS per year (OR = 15.46), and nocturnal seizures present (OR = 3.9). Patients with >3 GTCS per year (OR = 15.46) or nocturnal seizures (OR = 3.9) were classified as high risk. The OR cutoff value of 3.9 for the high-risk label was selected based on a previous SUDEP neuroimaging study (14), in which 90% of SUDEP cases were correctly identified as high risk if their summed OR score was at least 3.9 (presence of nocturnal seizures). Therefore, any patients above 3.9 were classed as “high risk” and any below were classed as “low risk.” In our cohort, this classification resulted in 14 high-risk (8 L TLE, 7 females) and 18 low-risk (9 L TLE, 9 females) subjects. Patient characteristics are shown in Table 1. There were no significant differences in the number of patients using multiple antiepileptic drugs (AEDs) (polytherapy) or one AED (monotherapy) between the high- and low-risk group. The average number of AEDs per high- and low-risk group was 2.4 and 2.2 respectively. AED dosages per high- and low-risk patients can be found in supplementary material (Table S1 in Supplementary Material).

**Table 1:** Summary of patients at low and high risk of SUDEP.

Characteristics	Low risk (*n* = 18)	High risk (*n* = 14)	*p*
Mean age at scan (years) ± SD	30.0 ± 7.1	33.5 ± 9.1	0.332
Gender (M:F)	9:9	7:7	1
Epilepsy lateralization (L:R)	9:9	8:6	0.693
Mean age epilepsy onset (years) ± SD	12.9 ± 9.5	12.4 ± 8.5	0.203
Mean epilepsy duration (years) ± SD	17.6 ± 10.3	21.2 ± 12.3	0.068
>3 GTCS per year	0	14	<0.001
Mean number of GTCS per year	0.3 ± 0.6	62 ± 58	<0.001
Nocturnal seizures	0	6	0.002
Hippocampal sclerosis	7	9	0.161
Polytherapy	13	12	0.367
Monotherapy	5	2	0.367
Mean SUDEP risk (OR) score ± SD	1.6 ± 1.7	19.2 ± 2.2	<0.001

*SUDEP, sudden death in epilepsy; L, left; R, right; GTCS, generalized tonic–clonic seizures; OR, odds ratio; AED, antiepileptic drug*.

### Functional MRI

All subjects underwent a 20-min resting-state electroencephalogram-functional magnetic resonance imaging (EEG-fMRI) scan (3.0 T scanner, Signa Excite HDX, GE Medical Systems), during which they were instructed to lay idly with eyes closed. The echo planar imaging-based blood oxygen level-dependent (BOLD) functional MRI scans were acquired with the following parameters: repetition time = 3,000 ms, echo time = 30 ms; flip angle = 90°, matrix size = 64 × 64, field of view = 24 cm × 24 cm, slice thickness = 3 mm, number of slices = 44. Simultaneous EEGs with 32 channels recorded with MRI compatible electrodes were acquired (Brain Products, Munich, Germany). The EEG recordings were used to exclude patients with epileptiform activity during the scan. The study was approved by the National Research Ethics Committee (04/Q0512/77 and 14/SW/0021) and all patients gave written informed consent.

### Data Preprocessing

The rs-fMRI time-series data were preprocessed in MATLAB 2016a (MathWorks Inc.) with DPARSFA (data processing assistant for resting state fMRI (37)) software, which calls functions from the software packages REST (38) and SPM12 (statistical parametric mapping; http://www.fil.ion.ucl.ac.uk/spm). The following steps were carried out: slice time correction, realignment, coregistration of structural and functional MRI images, segmentation *via* diffeomorphic anatomical registration through exponential lie algebra [DARTEL (39)], and spatial normalization to Montreal Neurological Institute space.

To reduce the effects of physiological noise, and to improve the specificity of signals pertaining to GM, the white matter (WM), and cerebrospinal fluid (CSF) signals were regressed out using the components analysis-based noise correction method, CompCor (40), in which five principal components derived from noise ROIs based on each subject’s segmented WM and CSF mask (mask threshold = 0.99) were removed (41).

The six motion realignment parameters calculated by SPM12 were also regressed out. Four patients from the original cohort were excluded due to maximum head motion exceeding 2 mm in any given direction of the rotation or translation parameters computed during realignment. For the remaining subjects included for further analysis, maximum head motion was 1.9 and 1.7 (mm) in the high- and low-risk groups, respectively. There were no significant differences in any motion parameters between the high- and low-risk cohorts (*p* > 0.05) as evaluated with independent samples *t*-tests. Head motion “scrubbing” was implemented, using DPARSFA’s built-in functions, to account for small but excessive head movements which are known to effect interregional correlations despite routine motion correction (42–46). For every scan in a given time series, the frame-wise displacement (FD), an index of head-movement from one volume to the next, was calculated as the sum of the absolute values of the realignment estimates relative to the preceding scan (42). Mean FD in the high- and low-risk cohorts was 0.15 ± 0.09 and 0.17 ± 0.08, respectively, and did not significantly differ (*t* = 0.586, *p* = 0.562). Scans to be scrubbed were defined as those for which FD exceeded 0.25 mm; for each of those, the preceding 1 and subsequent 2 scans were replaced *via* linear interpolation. In 5 of the original 60 patients, this procedure resulted in 75%, or more, of the scans being scrubbed—these patients were excluded from further analysis. In the remaining datasets, the proportion of scrubbed scans was below 50%. Finally, the linear trend was removed, and a bandpass filter of 0.01–0.08 Hz was applied, which is consistent with the frequency range most relevant to BOLD signal fluctuations. Spatial smoothing was not applied to not extend the BOLD signal between nearby ROIs.

### ROI Selection

**Figure 1:**
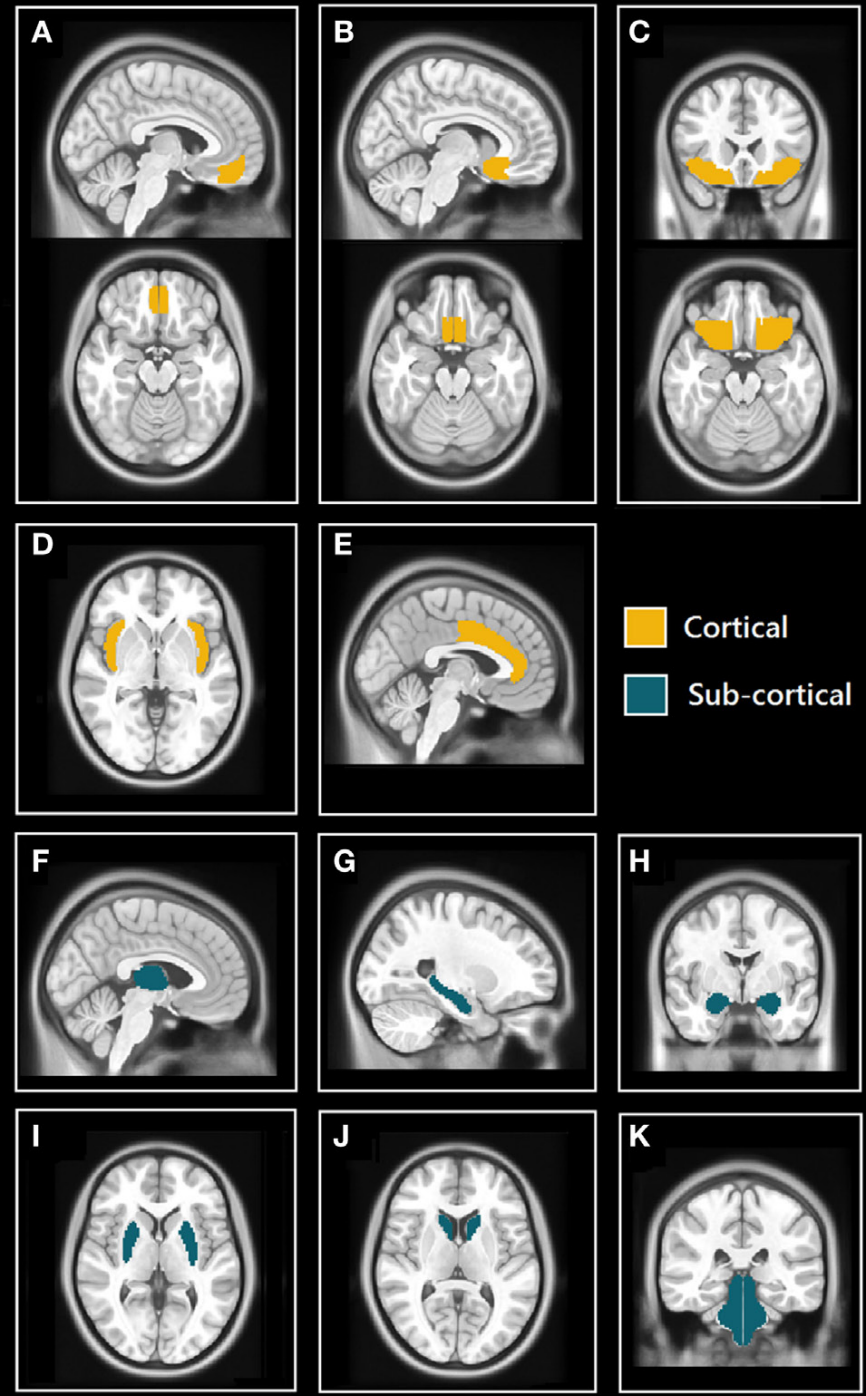
Selected cortical and subcortical regions of interest (ROIs) masks from Harvard-Oxford (HO) atlas. **(A)** Sagittal (top) and axial (bottom) views of the frontal medial cortex (FMC); **(B)** sagittal (top) and axial (bottom) views of the subcallosal cortex (SC); **(C)** coronal (top) and axial (bottom) views of orbitofrontal cortex (OFC); **(D)** axial view of insulae (Ins); **(E)** sagittal view of anterior cingulate cortex (ACC); **(F)** sagittal view of thalamus; **(G)** sagittal view of hippocampus; **(H)** coronal view of amygdalae; **(I)** axial view of putamen; **(J)** axial view of caudate; and **(K)** coronal view of brain stem (includes midbrain, pons, and medulla).

The Harvard-Oxford (HO) cortical and subcortical atlas (http://www.cma.mgh.harvard.edu/fsl_atlas.html) was used to extract ROI-averaged time-series from the processed fMRI time series. We selected 11 bilateral brain regions (22 total) from the HO atlas based on their known involvement in the central control of autonomic regulation (see Figure 1) These regions included structures belonging to the limbic system: hippocampus, amygdala, anterior cingulate cortex (ACC), and subcallosal cortex (SC); the insulae, thalamus, orbitofrontal cortex (OFC), frontal medial cortex (FMC), brain stem, and two regions of the basal ganglia: caudate and putamen.

### Resting-State FC Analysis and Network Based Statistic (NBS)

After extracting the time-series belonging to each ROI (network node), the absolute value of the Pearson *r* correlation coefficient was calculated for every possible ROI pair (each ROI pair defining a network edge or “path” between two structures) and a Fisher *Z*-transform normalization applied, yielding a 22 × 22 FC matrix for every subject. We then used the Network Based Statistic [NBS (35)] to compare the FC strength of every edge in the matrices between high risk and low risk of SUDEP patients. We sought to identify increased and decreased FC (contrasts: high risk < low risk; high risk > low risk) using analysis of covariance, with the following covariates: age, gender, lateralization of epilepsy, and presence of hippocampal sclerosis (HS). In addition to using presence of HS as a covariate, we also performed analyses whereby hippocampal GM volume of the epileptogenic hemisphere was regressed out (see Methods in Supplementary Material) in order to quantitatively control for differences in connectivity which may arise from changes in brain structure—namely, those resulting from HS.

In summary, NBS consists of the following steps: independently test the null hypothesis at every connection in the network using a two-sample *t*-test, endowing each edge with a *t*-statistic. A *t*-statistic threshold is required and must be specified prior to testing. Any edges for which the *t*-statistic threshold is exceeded are defined as suprathresholded connectivity. Clusters, or any set of nodes between which a path can be found, are then identified among the suprathresholded connectivity. The main assumption of the NBS is that any suprathresholded edges which form a cluster are not isolated from each other and therefore comprise a connected component, or subnetwork, differentiating the two groups (35). Finally, a family-wise error rate (FWER)-corrected *p* value is calculated using permutation testing (47). For each permutation, members of the two samples are randomly permuted, and the size of the extended cluster is calculated—in the current study, the number of permutations used was 10,000. These calculations yield an empirical null distribution of the maximal supra-threshold cluster size. Significance level was set at *p* < 0.05.

## Results

**Figure 2:**
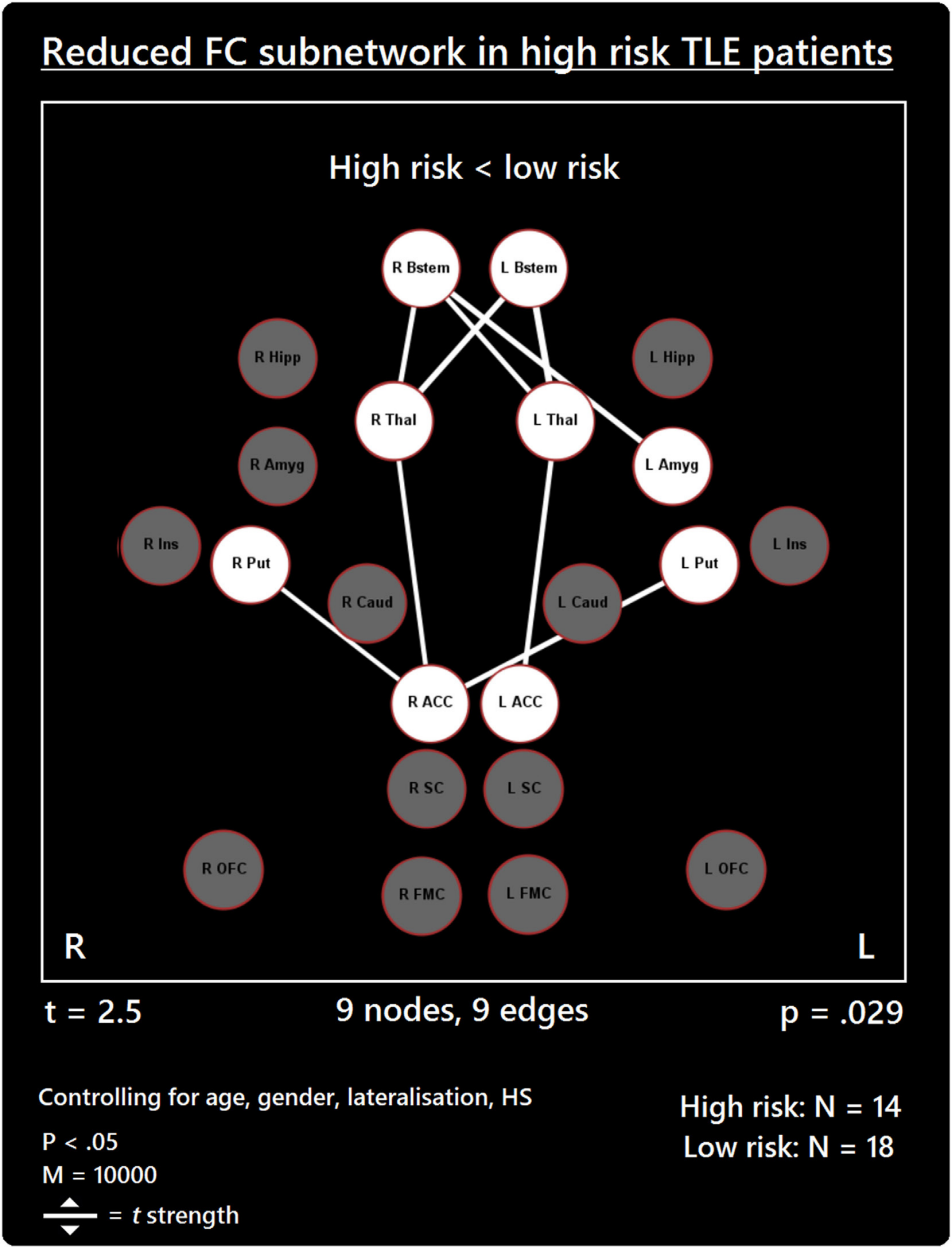
Reduced functional connectivity (FC) subnetwork in high risk over lower risk of sudden unexpected death in epilepsy (SUDEP) patients. Subnetwork of reduced FC involving the bilateral brain stem (Bstem), bilateral thalamus (Thal), bilateral putamen (Put), bilateral ACC, and left amygdala (Amyg). L, left; r, right; HS, hippocampal sclerosis; *t, t*-statistic threshold; *M*, number of permutations; *p* value was set at <0.05, family-wise error rate (FWER) corrected. Nodes in white are those which were involved in the significant subnetwork. Red node outline represents search for reduced connectivity (high < low). Visualization using Gephi (https://gephi.org/).

**Figure 3:**
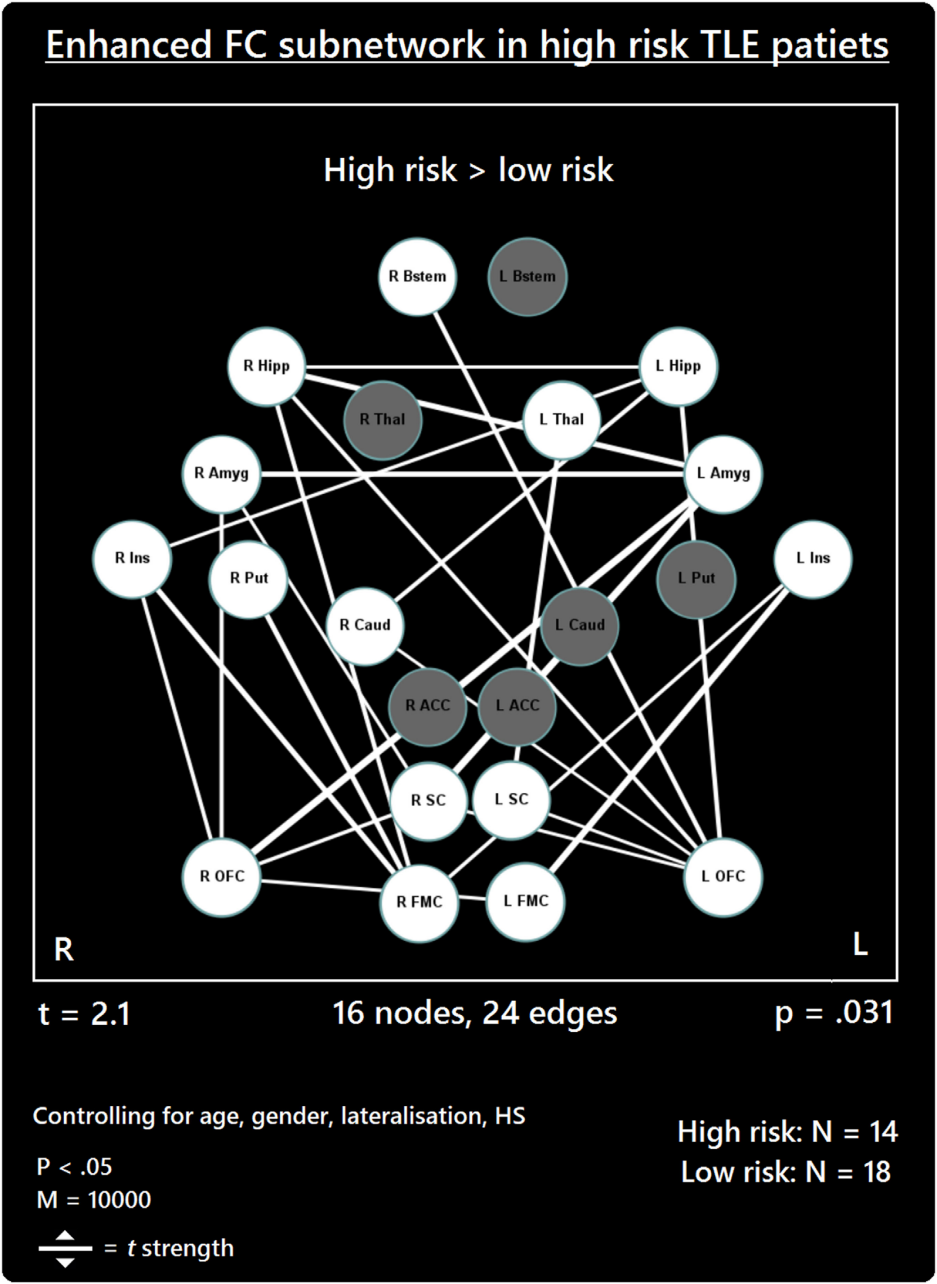
Increased functional connectivity (FC) subnetwork in high risk over lower risk of sudden unexpected death in epilepsy (SUDEP) patients. Subnetwork of enhanced FC in high-risk temporal lobe epilepsy (TLE) patients when compared with low-risk TLE patients. Regions include: bilateral amygdala (L Amyg, R Amyg), right brain stem (R Bstem), right caudate (R Caud), bilateral frontal medial cortex (L FMC, R FMC), bilateral hippocampus (L Hipp, R Hipp), bilateral insula (L Ins, R Ins), bilateral orbitofrontal cortex (L OFC, R OFC), right putamen (R Put), bilateral subcallosal cortex (L SC, R SC), and the left thalamus (L Thal). L, left; R, right; HS, hippocampal sclerosis; *t, t*-statistic threshold; *M*, number of permutations; *p* value was set at <0.05, family-wise error rate (FWER) corrected. White nodes represent ROIs involving significant connections. Blue node outline represents search for increased connectivity (high > low). Visualization using Gephi (https://gephi.org/).

The comparison between high- and low-risk SUDEP patients revealed a subnetwork of significantly reduced FC (*t* = 2.5, *p* = 0.029) and one subnetwork of significantly enhanced FC (*t* = 2.1, *p* = 0.033). The reduced FC subnetwork consisted of nine edges between the following nine nodes: bilateral ACC, bilateral thalamus, bilateral brain stem, left amygdala, and bilateral putamen (Figure 2; Table 2). The subnetwork of enhanced FC consisted of 16 nodes (bilateral FMC, bilateral SC, bilateral OFC, bilateral insula, bilateral hippocampus, bilateral amygdala, right caudate, right putamen, right brain stem, and left thalamus) and 24 edges (Figure 3; Table 3). Comparable significant subnetworks emerged following regression of hippocampal GM volume (instead of “presence of HS”). The high-risk < low-risk contrast revealed a significantly reduced subnetwork of 11 nodes (bilateral brain stem, bilateral thalamus, left amygdala, right insula, bilateral ACC, bilateral putamen and right SC) and 14 edges (*t* = 2.5, *p* = 0.035). The high-risk > low-risk contrast showed a significantly enhanced subnetwork comprising 15 nodes (bilateral hippocampus, amygdala, putamen, insula, SC, orbitofrontal cortex, medial frontal cortex, and right caudate) and 27 edges (*t* = 2.5, *p* = 0.028) (Results and Figures S1 and S2 in Supplementary Material).

**Table 2:** Reduced subnetwork.

Connection	*t*
L ACC–L thalamus	2.91
L brain stem–L thalamus	3.76
L brain stem–R thalamus	3.38
R ACC–R thalamus	2.66
R ACC–L putamen	3.04
R ACC–R putamen	2.59
R brain stem–L amygdala	3.23
R brain stem–L thalamus	2.87
R brain stem–R thalamus	2.89

*List of decreased connections belonging to the subnetwork of reduced connectivity found (high risk < low risk), with a threshold of *t* = 2.5*.

*t, t-test statistic*.

**Table 3:** Enhanced subnetwork.

Connection	*t*
L amygdala–R amygdala	2.68
L FMC–R OFC	2.21
L OFC–L hipp	2.51
L OFC–R brain stem	2.58
L OFC–R caudate	2.10
L OFC–R hipp	2.42
L hipp–R hipp	2.16
L ins–L FMC	2.99
L ins–R FMC	2.29
L SC–L OFC	2.14
L SC–L thalamus	2.62
R caudate–L hipp	2.46
R FMC–R hipp	2.49
R FMC–R putamen	2.69
R OFC–L amygdala	3.19
R OFC–R amygdala	2.39
R hipp–L amygdala	2.78
R ins–L hipp	2.24
R ins–R FMC	2.66
R ins–R OFC	2.35
R SC–L amygdala	3.17
R SC–L OFC	2.15
R SC–R amygdala	2.18
R SC–R OFC	2.35

*List of enhanced connections found using high-risk > low-risk contrast, with a threshold of t = 2.1*.

*t, t-test statistic*.

## Discussion

We examined whether, and to what extent, the FC between a group of structures associated with autonomic and respiratory regulation differs between TLE patients at high and low risk of SUDEP. We found that high-risk TLE patients exhibit highly altered FC among important brain regions known to be involved in autonomic regulation, when compared with low-risk patients. A subnetwork of reduced FC became apparent and involved several areas previously linked to increased SUDEP risk (17), including the thalamus, brain stem, and ACC. However, here we show involvement of additional brain regions which have not been previously linked to FC investigations into SUDEP, including the bilateral putamen and left amygdala. Additionally, we show a subnetwork of enhanced FC in TLE patients at high risk of SUDEP, the connections of which extended to many of the regions in the subnetwork. A large proportion of enhanced connections involved medial/orbital frontal cortices, the insulae and limbic areas (amygdalae and hippocampus). The ACC was not involved in enhanced FC in high-risk TLE patients. These findings prompt the need for further investigations into these structures, given their known involvement in cortical/subcortical autonomic control functions, particularly those pertaining to blood pressure regulation.

### Reduced FC Subnetwork

Our findings further support the importance of altered ACC-thalamus and thalamus–brain stem connectivity in patients at high risk of SUDEP (17). The thalamus relays extensive information to and from cortical and subcortical brain sites. The posterior thalamus plays significant roles in oxygen sensing (19, 48) and in relaying afferent activity essential for breathing. A disruption of the thalamic-brain stem link, as shown here in high-risk SUDEP patients, is particularly concerning given the apparent involvement of respiratory failure in SUDEP (9). The reduced thalamic connectivity bares resemblance to a VBM study showing reduced GM volumes in the thalamus among high-risk subjects and SUDEP victims (14). The Wandschneider study, and others [e.g., Ref. (49)], revealed however, that injury to the posterior thalamus is common in epilepsy, and disease duration potentiates the extent of that damage. Such injury may predispose to a failure to recover from hypoxia accompanying ictal episodes.

Anterior cingulate cortex involvement in autonomic regulation is well documented with early stimulation studies demonstrating its role in blood pressure regulation (50). Neuroimaging studies corroborated these findings (51) and show consistent fMRI activation and deactivation patterns of ACC in association with heart rate changes (52, 53), and cold pressor and hand-grip responses (20). In human epilepsy, thalamic-cingulate circuitry alterations were previously described (17, 54). Upon stimulation of the cingulate, asystole—a potential SUDEP mechanism—has been observed (55). The reduced thalamic–ACC connectivity among patients at high risk for SUDEP reflects a disruption of key pathways involved in central modulation of cardiorespiratory and blood pressure mechanisms, which may be implicated in SUDEP (34).

Our data reveal for the first time a role of the putamen in the reduced connectivity subnetwork found in high-risk SUDEP patients. The putamen serves significant autonomic regulatory behaviors, and has major projections to insular and limbic sites (56, 57). The putamen also serves to integrate sensory information for preparation of movements (58, 59). Reduced connectivity between the putamen and ACC could alter communication between autonomic and motor regulatory pathways in patients at high risk of SUDEP. Furthermore, we show reduced FC of the bilateral putamen with the right ACC only. The right ACC is preferentially involved in baroreflex-mediated autonomic cardiovascular function in humans (60). Patients with congenital central hypoventilation syndrome (CCHS), who are also at high risk of sudden death, show BOLD signal reductions within the putamen when compared with controls (61).

Reduced FC between the right brain stem and left amygdala also occurred in high-risk SUDEP patients. The final common path nuclei for cardiac, respiratory, and blood pressure control lie within the brain stem. The involvement of the amygdala in cardiovascular and respiratory activities has been described (62, 63), as are the afferent and efferent pathways through which the amygdala projects to the midbrain, pons, and brain stem (22, 64–66). Single-pulse stimulation of the amygdala central nucleus will trigger state-dependent inspiration (67). Animal models reveal a vital role of the amygdala in the propagation of seizures from the brain stem to the forebrain (68). Upon stimulation of the amygdala in patients with epilepsy, apnea and oxygen desaturation are observed (8, 63, 69), demonstrating the significant influences of this structure on brain stem respiratory nuclei in humans. The reduced FC we found between the amygdala and brain stem in high-risk patients is of considerable concern, especially given the occurrence of terminal apnea in the majority of SUDEP cases (9). We speculate that the FC reduction may contribute to a failure of amygdala influences to trigger inspiratory efforts and recover from possible hypoventilation or apnea during seizures, or possibly result in sustained apneusis. Reduced FC may provide a marker for the respiratory alterations in epilepsy and mechanisms underpinning SUDEP.

### Enhanced FC Subnetwork

As well as reduced connectivity, an enhanced FC subnetwork emerged in high-risk SUDEP patients involving 24 increased functional links in 16 of the 22 regions investigated (Figure 1). The majority of enhanced connectivity patterns found in high-risk patients were represented by connections from the frontal medial or orbital frontal cortex to the insula and limbic cortices (hippocampus and amygdala).

The enhanced FC of orbital frontal and frontal medial cortices found here is of particular interest given their involvement in blood pressure modulation (62, 70). Portions of the medial prefrontal cortex also influence key areas involved in modulating cardiac sympathetic and parasympathetic responses and baroreflex activity (71–73). Hand grip tasks, which induce heart rate changes, are associated with reduced activity within the hippocampus, orbitofrontal and medial prefrontal cortex in human subjects (52, 60, 74), demonstrating the largely inhibitory role of the medial and orbital frontal cortices in autonomic regulation (21, 25, 60). The enhanced connectivity observed between medial prefrontal cortices and limbic structures in high-risk SUDEP patients could reflect an imbalance in the medial prefrontal–hippocampal circuitry involved in blood pressure regulation (18, 19). A recent rs-fMRI study showed that increased vagal modulation, as measured by postexercise HRV, is accompanied by increased FC between the right anterior hippocampus and the ventral medial prefrontal cortex (75). Of interest, enhanced FC between the right hippocampus and the right FMC emerged in our study, and not in the left in high-risk patients at rest. One possibility, therefore, is that this increased connectivity is linked to resting elevated sympathetic tone, which is exhibited among poorly controlled epilepsy patients (31).

The increased connectivity between the insulae and the OFC/FMC revealed in the enhanced subnetwork may be linked with blood pressure regulation during ictal periods. A role of the insula in autonomic regulation has long been known (76), with neuroimaging studies confirming earlier stimulation studies (77). The right insula exerts major influences over sympathetic control, while the left insula more-prominently influences parasympathetic outflow (76, 78, 79). The inhibitory role of the medial prefrontal cortex is well known for both autonomic processes and somatic reflexes (80). Increased activation of the insula, coupled with observed decreases in the medial prefrontal cortex (60, 74) during autonomic challenges implies a dynamic relationship between these regions which, along with the hippocampus, forms part of a major network concerned with blood pressure regulation (21). Projections from the insular cortex to the medial frontal cortex, if exaggerated by seizure discharge, could lead to enhanced suppression of blood pressure levels, with the potential for hypotension. Such an imbalance at rest provides evidence of dysfunctional networking among these structures which may alter their ability to recover following a significant disturbance, such as a seizure.

The current data also demonstrate increased FC between the left and right hippocampus and left and right amygdalae in high-risk patients. Human electrophysiological studies demonstrate homotopic connectivity of bilateral mesial temporal structures in drug-resistant focal epilepsy (81). Stimulation of the fornix results in contralateral hippocampal responses without involvement from the neocortex, establishing a link between bilateral mesial temporal structures (82). These findings also demonstrate that temporal lobe seizures likely propagate between the hemispheres *via* the limbic system. The high functional interconnectivity among high-risk patients between the bilateral amygdalae and bilateral hippocampi poses a risk of exaggerated descending influences on both breathing and blood pressure. The role of the amygdala in both sustaining inspiration (67) with the potential for apneusis or generating apnea has been described earlier (8, 69). If both amygdalae combine to exert influences to the phase-switching brain stem areas, the risk for apneusis or apnea is raised. The hippocampus plays an essential role in the diencephalic blood pressure regulatory circuitry (21, 62, 70). Safe constraints may exist with unilateral influences, but bilateral extreme activation, as may happen by recruitment in ictal discharge, may pose overwhelming drives to lower blood pressure final common path structures. Resting interictal imbalances as shown here could result in erroneous and disturbed autoregulation during extreme circumstances, such as during ictal or postictal periods. Given the much higher frequency of seizures experienced by the high-risk cohort, it is plausible to suggest that these enhanced connections may be evidence of long-term seizure-induced hyperconnectivity of these structures. However, further work is required to establish whether and how seizure frequency influences the homotopic connectivity of these, and other, structures in TLE and other epilepsies.

The SC exhibited increased FC with the amygdala, OFC, and thalamus. Recent investigations involving direct cortical stimulation of the SC in human epilepsy patients demonstrate its role in cortical control of blood pressure. Significant hypotensive changes were observed upon stimulation of the bilateral SC (Lacuey et al., Unpublished).^1^ These findings confirm subcallosal involvement in cortical blood pressure control, and implicate this region in the genesis of peri-ictal hypotension in epilepsy patients (see footnote text1). The increased connections found in high-risk patients indicate further hyperconnective imbalances among autonomic regions, particularly those involved in blood pressure regulation.

The basal ganglia participate heavily in autonomic regulation, which likely follows from its prominent projections to the lateral hypothalamus and nuclei in the brain stem (83). Deterioration of the basal ganglia has been linked to cardiovascular disturbances, particularly relating to blood pressure, observed in Parkinson’s disease (57, 84, 85). As well as receiving strong thalamic input, portions of the basal ganglia, particularly the caudate, share connectivity with cortical sites, including the medial/orbital frontal cortices and hippocampal and amygdala structures (86, 87). The basal ganglia sites are involved in the complex circuitry responsible for modulation of signaling between cortical and subcortical structures, and are linked with many other processes, including those related to arousal- (88), sensory- (89), and cognitive-based (90) functions. The enhanced connections involving the putamen and caudate among the subnetwork provide further evidence of the potential for disturbed communication between cortical and subcortical systems to exert profound autonomic distortions in patients who are at high risk of SUDEP.

### Results after Regression of Hippocampal GM Volume, Not Presence of HS

As well as using presence/non-presence of HS as a covariate, we conducted further analyses using a more quantitative approach to control for connectivity changes related to morphological differences of the mesial temporal structures (hippocampus) between high- and low-risk patients. Similar reduced and enhanced subnetworks were revealed following this approach and, importantly, the core effects observed using presence of HS as a covariate were mirrored in this analysis. In summary, these were: reduced connectivity of the brain stem, thalamus, amygdala and putamen; and enhanced connectivity involving medial and orbital frontal cortices, the insulae, hippocampi and amygdalae, putamen, and caudate (see Results and Figures S1 and S2 in Supplementary Material). Additional edges were revealed in the reduced subnetwork and comprised connections from the brain stem to the insula, putamen, and SC, and from the subcallosal to the ACC. In the high-risk > low-risk contrast, a greater number of connections involving the left medial frontal cortex emerged, and enhanced bilateral homotopic connectivity of the frontal medial and SC is shown. These connections highlight further altered connectivity in relation to increased SUDEP risk which must be explored in future studies. These data also demonstrate the importance of taking into account volumetric alterations in connectivity analyses, which should be considered in future studies.

### Neuroimaging Findings in Other Cohorts at Risk of Sudden Death

Alterations in brain structure, function, and connectivity are not unique to subjects with epilepsy at risk for SUDEP. Neuroimaging studies of other syndromes in which risk of sudden death is high reveal both structural and functional brain alterations between autonomic regulatory brain areas in patient groups compared with controls. Heart failure (HF) patients show damage to cortical autonomic regions, including the insulae, anterior cingulate, subgenu, and the ventromedial prefrontal cortex (VMPFC) (91), as well as volume loss in the putamen (92). Obstructive sleep apnea (OSA) subjects show significant amplitude and phase changes in functional MRI signals of autonomic and respiratory regulatory structures to blood pressure and ventilator challenges [for reviews see Ref. (93) or (94)], as well as highly altered FC of the insular cortices in patients (95) and volumetric alterations of the putamen (96). Patients with congenital central hypoventilation syndrome (CCHS), a syndrome accompanied by severe disturbances in both autonomic and respiratory function (97), show cortical thinning of the insular cortex, cingulate, and VMPFC (19, 98–100), as well as injury to hippocampal and other limbic structures (19). These syndromes, especially heart failure and CCHS, share a risk for sudden, unexpected death with the epilepsy group studied here, especially during sleep. Moreover, both structural injury and fMRI signal responses to challenges were lateralized in these other conditions.

### Known Interictal Autonomic Disturbances in Epilepsy and Relation to the Current Findings

Temporal lobe epilepsy patients show highly altered interictal HRV (101) which reflect imbalances in sympathetic and parasympathetic control over cardiorespiratory actions, and is particularly disturbed in refractory epilepsy patients and those who experience GTCS (31, 32). Increased SUDEP risk has been associated with such alterations in HRV (33), particularly reductions in root-mean square differences of successive R-R intervals (102)—a measure of HRV which reflects vagus nerve-meditated autonomic control of the heart (33). The findings outlined in the current study may shed light on the underlying neural correlates of such autonomic imbalances in TLE patients at high risk of SUDEP.

### Limitations and Future Work

#### ROI Issues and Considerations

A potential drawback of the current study is the incomplete parcelation of the template used to define ROIs. Many of the structures investigated here contain subdivisions which may be important for interpreting the relevance of our findings with respect to their specific autonomic function. For example, the insular cortices are large structures, the subdivisions of which have differential roles in autonomic function (79). The lack of insular subdivisions in the current study hampers interpretation of enhanced connections found involving this structure. Similarly, subdivisions of the hippocampus also serve different functions (103), and future brain stem studies should include, at least, separation of the midbrain, pons, and medulla. The thalamus also contains multiple subdivisions, each with specialized functions and which project to different sites (104). Portions of the posterior thalamus, for example, play a critical role in oxygen and CO_2_ regulation (19, 48), and the region shows reduced GM volume among high-risk patients and SUDEP victims (14) and is also damaged in CCHS patients (19).

The current study did not consider the cerebellum among the selected ROIs due to inadequate scan coverage. The cerebellum has been extensively linked to autonomic and respiratory functions, and especially with its role in dampening extremes of blood pressure changes (19), and is another structure which exhibits damage in HF patients, who are at considerable risk of sudden death (105). Exploring functional interactions between the cerebellum and other brain structures in epilepsy, and with particular respect to SUDEP, is of significant interest. Future studies investigating structural and functional changes in this setting should include both cerebellar cortex and deep “autonomic” nuclei in the evaluation.

#### Network-Based Statistic Limitations and Choice of Statistical Significance Threshold

The NBS enables detection of cluster-based differences (components) among a set of connections (in a network), enabling differentiation of two group-based significant subnetworks. Thus, the NBS has reduced power to detect stand-alone connections as belonging to the significant detected component. Furthermore, identification of a cluster relies first, on detection of edges which surpass a given threshold (*t*), which must be specified *a priori*. One drawback of this approach is that it is rarely known which *t* should be used in practice, resulting in an unavoidable level of subjectivity. To limit this bias here, we chose the minimum threshold at which a significant subnetwork for each contrast was revealed. The threshold required to reveal the reduced subnetwork (high risk < low risk) was *t* = 2.5, while *t* = 2.1 was required to reveal the enhanced subnetwork. The relatively higher threshold used in the high-risk < low-risk contrast reflects the discovery of a smaller, but more intense subnetwork of reduced FC, while the slightly lower threshold used for the high-risk > low-risk contrast explains the more extended yet less intense subnetwork of increased FC found (35).

#### Cohort

Future neuroimaging studies investigating SUDEP would benefit from applying network-based FC approaches to larger samples involving more epilepsy subtypes and patients who are subsequent victims of SUDEP. Furthermore, comparisons involving a group of healthy subjects are also necessary to evaluate findings in patients with reference to the healthy brain. Further sampling issues relate to inclusion of left and right TLE patients in the same group which, although controlled in statistical analysis, does not offer the opportunity to independently explore high-risk vs. low-risk differences in each subgroup separately. Such an investigation would be of interest, given the lateralization of autonomic brain circuitry (20) and the known whole-brain network differences between left and right TLE patients (106).

Neuroimaging studies have demonstrated altered FC of mesial temporal structures, including the hippocampus and amygdala (107), and medial prefrontal regions, including the SC (108), among patients with depression. Given the overlap involving epilepsy and psychiatric complications such as depression and anxiety (109), future efforts should include methods to partition variance due to the incidence and severity of psychiatric diagnoses.

Finally, while the current study offers insight into connectivity differences among cortical and subcortical autonomic regions at rest, it would be of interest to evaluate functional responses in relation to task-based fMRI assessments of autonomic and respiratory brain function in epilepsy patients. Particularly important would be comparisons between patients who with and without GTCS. Investigating activation patterns in direct association with autonomic and respiratory challenges could shed light on affected autonomic brain function as a function of GTCS frequency—the most significant SUDEP risk factor (11).

## Conclusion

Alterations in FC observed indicate a dysfunctional network of critical cortical and subcortical brain regions involved in autonomic and respiratory regulation. Resting-state FC imbalances among these regulatory structures may predispose such a network to fail to recover from extremities caused by seizures, particularly GTCS. Our results build on existing findings and shed further light on interactions between affected structures related to increased SUDEP risk and underline the importance of laterality considerations on connectivity, and the need to consider integration from multiple brain sites in evaluating autonomic or breathing outcomes in SUDEP mechanisms.

## Ethics Statement

Informed written consent was obtained from all participants. The study was approved by the Health Research Authority (HRA)—NHS England.

## Author Contributions

LA prepared and analyzed data. LA, RH, RK, LL, and BD wrote the manuscript. All authors contributed editorially. RH, RK, JO, and MG advised on ROI selection, imaging analysis and interpretation of findings. RH, BD, SL, and SR helped to refine the clinical and physiological interpretation of findings. CS advised on clinical and neurophysiological issues. MG, SV, and LL advised on imaging and methodological issues.

## Conflict of Interest Statement

The authors declare that the research was conducted in the absence of any commercial or financial relationships that could be construed as a potential conflict of interest.

## Footnotes

^1^Lacuey N, Hampson JP, Theeranaew W, Zonjy B, Vithala A, Loparo K, et al. Brodmann area 25 is a cortical structure for human blood pressure control.

## References

[1] Tomson T Nashef L Ryvlin P. Sudden unexpected death in epilepsy: current knowledge and future directions. Lancet Neurol (2008) 7(11):1021–31. 10.1016/S1474-4422(08)70202-3 18805738

[2] Flicker DM So EL Shen WK Annegers JF O’Brien PC Cascino GD Population-based study of the incidence of sudden unexplained death in epilepsy. Neurology (1998) 51(5):1270–4. 10.1212/WNL.51.5.1270 9818844

[3] Surges R Sander JW. Sudden unexpected death in epilepsy: mechanisms, prevalence, and prevention. Curr Opin Neurol (2012) 25(2):201–7. 10.1097/WCO.0b013e3283506714 22274774

[4] Devinsky O Sudden, unexpected death in epilepsy. N Engl J Med (2011) 365(19):1801–11. 10.1056/NEJMra1010481 22070477

[5] Nashef L Hindocha N Makoff A. Risk factors in sudden death in epilepsy (SUDEP): the quest for mechanisms. Epilepsia (2007) 48(5):859–71. 10.1111/j.1528-1167.2007.01082.x 17433051

[6] Surges R Thijs RD Tan HL Sander JW. Sudden unexpected death in epilepsy: risk factors and potential pathomechanisms. Nat Rev Neurol (2009) 5(9):492–504. 10.1038/nrneurol.2009.118 19668244

[7] Massey CA Sowers LP Dlouhy BJ Richerson GB. Mechanisms of sudden unexpected death in epilepsy: the pathway to prevention. Nat Rev Neurol (2014) 10(5):271–82. 10.1038/nrneurol.2014.64 24752120 PMC4565133

[8] Dlouhy BJ Gehlbach BK Richerson GB Sudden unexpected death in epilepsy: basic mechanisms and clinical implications for prevention. J Neurol Neurosurg Psychiatry (2015):1–12. 10.1136/jnnp-2013-307442 26979537

[9] Ryvlin P Nashef L Lhatoo SD Bateman LM Bird J Bleasel A Incidence and mechanisms of cardiorespiratory arrests in epilepsy monitoring units (MORTEMUS): a retrospective study. Lancet Neurol (2013) 12(10):966–77. 10.1016/S1474-4422(13)70214-X 24012372

[10] Shorvon S Tomson T Sudden unexpected death in epilepsy. Lancet (2011) 378(9808):2028–38. 10.1016/S0140-6736(11)60176-1 21737136

[11] Hesdorffer DC Tomson T Benn E Sander JW Nilsson L Langan Y Combined analysis of risk factors for SUDEP. Epilepsia (2011) 52(6):1150–9. 10.1111/j.1528-1167.2010.02952.x 21671925

[12] Harden C Tomson T Gloss D Buchhalter J Cross JH Donner E Practice guideline summary: sudden unexpected death in epilepsy incidence rates and risk factors Report of the Guideline Development, Dissemination, and Implementation Subcommittee of the American Academy of Neurology and the American Epilepsy Society. Neurology (2017) 88(17):1674–80. 10.1212/WNL.0000000000003685 28438841

[13] Lamberts RJ Thijs RD Laffan A Langan Y Sander JW. Sudden unexpected death in epilepsy: people with nocturnal seizures may be at highest risk. Epilepsia (2012) 53(2):253–7. 10.1111/j.1528-1167.2011.03360.x 22192074

[14] Wandschneider B Koepp M Scott C Micallef C Balestrini S Sisodiya SM Structural imaging biomarkers of sudden unexpected death in epilepsy. Brain (2015) 138(10):2907–19. 10.1093/brain/awv233 26264515 PMC4671481

[15] Mueller SG Bateman LM Laxer KD. Evidence for brainstem network disruption in temporal lobe epilepsy and sudden unexplained death in epilepsy. Neuroimage Clin (2014) 5:208–16. 10.1016/j.nicl.2014.06.010 25068110 PMC4110882

[16] Greicius MD Krasnow B Reiss AL Menon V Functional connectivity in the resting brain: a network analysis of the default mode hypothesis. Proc Natl Acad Sci U S A (2003) 100(1):253–8. 10.1073/pnas.0135058100 12506194 PMC140943

[17] Tang Y Chen Q Yu X Xia W Luo C Huang X A resting-state functional connectivity study in patients at high-risk for sudden unexpected death in epilepsy. Epilepsy Behav (2014) 41:33–8. 10.1016/j.yebeh.2014.08.140 25277976

[18] Shoemaker JK Goswami R. Forebrain neurocircuitry associated with human reflex cardiovascular control. Front Physiol (2015) 6:240. 10.3389/fphys.2015.00240 26388780 PMC4555962

[19] Harper RM Kumar R Macey PM Harper RK Ogren JA. Impaired neural structure and function contributing to autonomic symptoms in congenital central hypoventilation syndrome. Front Neurosci (2015) 9:415. 10.3389/fnins.2015.00415 26578872 PMC4626648

[20] Macey PM Ogren JA Kumar R Harper RM. Functional imaging of autonomic regulation: methods and key findings. Front Neurosci (2016) 9:513. 10.3389/fnins.2015.00513 26858595 PMC4726771

[21] Shoemaker JK Norton KN Baker J Luchyshyn T. Forebrain organization for autonomic cardiovascular control. Auton Neurosci (2015) 188:5–9. 10.1016/j.autneu.2014.10.022 25458433

[22] Hopkins DA Holstege G. Amygdaloid projections to the mesencephalon, pons and medulla oblongata in the cat. Exp Brain Res (1978) 32(4):529–47. 10.1007/BF00239551 689127

[23] Holstege G Meiners L Tan K. Projections of the bed nucleus of the stria terminalis to the mesencephalon, pons, and medulla oblongata in the cat. Exp Brain Res (1985) 58(2):379–91. 10.1007/BF00235319 3996501

[24] Verberne AJ Owens NC. Cortical modulation of the cardiovascular system. Prog Neurobiol (1998) 54(2):149–68. 10.1016/S0301-0082(97)00056-7 9481796

[25] Owens NC Verberne AJ. Medial prefrontal depressor response: involvement of the rostral and caudal ventrolateral medulla in the rat. J Auton Nerv Syst (2000) 78(2):86–93. 10.1016/S0165-1838(99)00062-4 10789686

[26] Wilson CL Isokawa M Babb TL Crandall PH Functional connections in the human temporal lobe. Exp Brain Res (1990) 82(2):279–92. 10.1007/BF00231248 2286232

[27] Rugg-Gunn FJ Holdright D Epilepsy and the heart. Br J Cardiol (2010) 17(5):223–9.

[28] Devinsky O. Effects of seizures on autonomic and cardiovascular function. Epilepsy Curr (2004) 4(2):43–6. 10.1111/j.1535-7597.2004.42001.x 15562299 PMC531654

[29] Opherk C Coromilas J Hirsch LJ. Heart rate and EKG changes in 102 seizures: analysis of influencing factors. Epilepsy Res (2002) 52(2):117–27. 10.1016/S0920-1211(02)00215-2 12458028

[30] Leutmezer F Schernthaner C Lurger S Pötzelberger K Baumgartner C. Electrocardiographic changes at the onset of epileptic seizures. Epilepsia (2003) 44(3):348–54. 10.1046/j.1528-1157.2003.34702.x 12614390

[31] Mukherjee S Tripathi M Chandra PS Yadav R Choudhary N Sagar R Cardiovascular autonomic functions in well-controlled and intractable partial epilepsies. Epilepsy Res (2009) 85(2):261–9. 10.1016/j.eplepsyres.2009.03.021 19409754

[32] Evrengül H Tanriverdi H Dursunoglu D Kaftan A Kuru O Unlu U Time and frequency domain analyses of heart rate variability in patients with epilepsy. Epilepsy Res (2005) 63(2):131–9. 10.1016/j.eplepsyres.2005.02.001 15777689

[33] DeGiorgio CM Miller P Meymandi S Chin A Epps J Gordon S RMSSD, a measure of vagus-mediated heart rate variability, is associated with risk factors for SUDEP: the SUDEP-7 Inventory. Epilepsy Behav (2010) 19(1):78–81. 10.1016/j.yebeh.2010.06.011 20667792 PMC2943000

[34] Bozorgi A Chung S Kaffashi F Loparo KA Sahoo S Zhang GQ Significant postictal hypotension: expanding the spectrum of seizure-induced autonomic dysregulation. Epilepsia (2013) 54(9):e127–30. 10.1111/epi.12251 23758665 PMC3769446

[35] Zalesky A Fornito A Bullmore ET. Network-based statistic: identifying differences in brain networks. Neuroimage (2010) 53(4):1197–207. 10.1016/j.neuroimage.2010.06.041 20600983

[36] Nichols TE Holmes AP. Nonparametric permutation tests for functional neuroimaging: a primer with examples. Hum Brain Mapp (2002) 15(1):1–25. 10.1002/hbm.1058 11747097 PMC6871862

[37] Yan C Zang Y DPARSF: a MATLAB toolbox for” pipeline” data analysis of resting-state fMRI. Front Syst Neurosci (2010) 4:13. 20577591 10.3389/fnsys.2010.00013 PMC2889691

[38] Song XW Dong ZY Long XY Li SF Zuo XN Zhu CZ REST: a toolkit for resting-state functional magnetic resonance imaging data processing. PLoS One (2011) 6(9):e25031. 10.1371/journal.pone.0025031 21949842 PMC3176805

[39] Ashburner J. A fast diffeomorphic image registration algorithm. Neuroimage (2007) 38(1):95–113. 10.1016/j.neuroimage.2007.07.007 17761438

[40] Behzadi Y Restom K Liau J Liu TT. A component based noise correction method (CompCor) for BOLD and perfusion based fMRI. Neuroimage (2007) 37(1):90–101. 10.1016/j.neuroimage.2007.04.042 17560126 PMC2214855

[41] Muschelli J Nebel MB Caffo BS Barber AD Pekar JJ Mostofsky SH. Reduction of motion-related artifacts in resting state fMRI using aCompCor. Neuroimage (2014) 96:22–35. 10.1016/j.neuroimage.2014.03.028 24657780 PMC4043948

[42] Power JD Barnes KA Snyder AZ Schlaggar BL Petersen SE. Spurious but systematic correlations in functional connectivity MRI networks arise from subject motion. Neuroimage (2012) 59(3):2142–54. 10.1016/j.neuroimage.2011.10.018 22019881 PMC3254728

[43] Satterthwaite TD Wolf DH Loughead J Ruparel K Elliott MA Hakonarson H Impact of in-scanner head motion on multiple measures of functional connectivity: relevance for studies of neurodevelopment in youth. Neuroimage (2012) 60(1):623–32. 10.1016/j.neuroimage.2011.12.063 22233733 PMC3746318

[44] Van Dijk KR Sabuncu MR Buckner RL. The influence of head motion on intrinsic functional connectivity MRI. Neuroimage (2012) 59(1):431–8. 10.1016/j.neuroimage.2011.07.044 21810475 PMC3683830

[45] Power JD Mitra A Laumann TO Snyder AZ Schlaggar BL Petersen SE. Methods to detect, characterize, and remove motion artifact in resting state fMRI. Neuroimage (2014) 84:320–41. 10.1016/j.neuroimage.2013.08.048 23994314 PMC3849338

[46] Lemieux L Salek-Haddadi A Lund TE Laufs H Carmichael D. Modelling large motion events in fMRI studies of patients with epilepsy. Magn Reson Imaging (2007) 25(6):894–901. 10.1016/j.mri.2007.03.009 17490845

[47] Freedman D Lane D A nonstochastic interpretation of reported significance levels. J Bus Econ Stat (1983) 1(4):292–8. 10.2307/1391660

[48] Koos BJ Chau A Matsuura M Punla O Kruger L. Thalamic locus mediates hypoxic inhibition of breathing in fetal sheep. J Neurophysiol (1998) 79(5):2383–93. 9582214 10.1152/jn.1998.79.5.2383

[49] Keller SS Wieshmann UC Mackay CE Denby CE Webb J Roberts N Voxel based morphometry of grey matter abnormalities in patients with medically intractable temporal lobe epilepsy: effects of side of seizure onset and epilepsy duration. J Neurol Neurosurg Psychiatry (2002) 73(6):648–55. 10.1136/jnnp.73.6.648 12438464 PMC1757338

[50] Pool JL Ransohoff J Autonomic effects on stimulating rostral portion of cingulate gyri in man. J Neurophysiol (1949) 12(6):385–92. 15408005 10.1152/jn.1949.12.6.385

[51] Macefield VG Henderson LA. Autonomic responses to exercise: cortical and subcortical responses during post-exercise ischaemia and muscle pain. Auton Neurosci (2015) 188:10–8. 10.1016/j.autneu.2014.10.021 25458426

[52] Shoemaker JK Wong SW Cechetto DF Cortical circuitry associated with reflex cardiovascular control in humans: does the cortical autonomic network “speak” or “listen” during cardiovascular arousal. Anat Rec (2012) 295(9):1375–84. 10.1002/ar.22528 22848047

[53] Critchley HD Mathias CJ Josephs O O’Doherty J Zanini S Dewar BK Human cingulate cortex and autonomic control: converging neuroimaging and clinical evidence. Brain (2003) 126(10):2139–52. 10.1093/brain/awg216 12821513

[54] Bonilha L Rorden C Castellano G Cendes F Li LM. Voxel-based morphometry of the thalamus in patients with refractory medial temporal lobe epilepsy. Neuroimage (2005) 25(3):1016–21. 10.1016/j.neuroimage.2004.11.050 15809001

[55] Leung H Schindler K Kwan P Elger C. Asystole induced by electrical stimulation of the left cingulate gyrus. Epileptic Disord (2007) 9(1):77–81. 10.1684/epd.2007.0054 17307716

[56] Saper CB. Convergence of autonomic and limbic connections in the insular cortex of the rat. JComp Neurol (1982) 210(2):163–73. 10.1002/cne.902100207 7130477

[57] Pazo JH Belforte JE. Basal ganglia and functions of the autonomic nervous system. Cell Mol Neurobiol (2002) 22(5):645–54. 10.1023/A:1021844605250 12585684 PMC11533747

[58] Alexander GE Crutcher MD. Functional architecture of basal ganglia circuits: neural substrates of parallel processing. Trends Neurosci (1990) 13(7):266–71. 10.1016/0166-2236(90)90107-L 1695401

[59] Marchand WR Lee JN Thatcher JW Hsu EW Rashkin E Suchy Y Putamen coactivation during motor task execution. Neuroreport (2008) 19(9):957–60. 10.1097/WNR.0b013e328302c873 18521000

[60] Kimmerly DS O’Leary DD Menon RS Gati JS Shoemaker JK. Cortical regions associated with autonomic cardiovascular regulation during lower body negative pressure in humans. J Physiol (2005) 569(1):331–45. 10.1113/jphysiol.2005.091637 16150800 PMC1464214

[61] Macey PM Woo MA Macey KE Keens TG Saeed MM Alger JR Hypoxia reveals posterior thalamic, cerebellar, midbrain, and limbic deficits in congenital central hypoventilation syndrome. J Appl Physiol (2005) 98(3):958–69. 10.1152/japplphysiol.00969.2004 15531561

[62] Harper RM Gozal D Bandler R Spriggs D Lee J Alger J. Regional brain activation in humans during respiratory and blood pressure challenges. Clin Exp Pharmacol Physiol (1998) 25(6):483–6. 10.1111/j.1440-1681.1998.tb02240.x 9673830

[63] Lacuey N Zonjy B Londono L Lhatoo SD. Amygdala and hippocampus are symptomatogenic zones for central apneic seizures. Neurology (2017) 88(7):701–5. 10.1212/WNL.0000000000003613 28087822 PMC5317387

[64] Saper CB Loewy AD. Efferent connections of the parabrachial nucleus in the rat. Brain Res (1980) 197(2):291–317. 10.1016/0006-8993(80)91117-8 7407557

[65] Usunoff KG Itzev DE Rolfs A Schmitt O Wree A. Brain stem afferent connections of the amygdala in the rat with special references to a projection from the parabigeminal nucleus: a fluorescent retrograde tracing study. Anat Embryol (2006) 211(5):475–96. 10.1007/s00429-006-0099-8 16763808

[66] Price JL Drevets WC. Neurocircuitry of mood disorders. Neuropsychopharmacology (2010) 35(1):192–216. 10.1038/npp.2009.104 19693001 PMC3055427

[67] Harper RM Frysinger RC Trelease RB Marks JD. State-dependent alteration of respiratory cycle timing by stimulation of the central nucleus of the amygdala. Brain Res (1984) 306:1–8. 10.1016/0006-8993(84)90350-0 6466967

[68] Hirsch E Danober L Simler S De Vasconcelos AP Maton B Nehlig A The amygdala is critical for seizure propagation from brainstem to forebrain. Neuroscience (1997) 77(4):975–84. 10.1016/S0306-4522(96)00503-9 9130779

[69] Dlouhy BJ Gehlbach BK Kreple CJ Kawasaki H Oya H Buzza C Breathing inhibited when seizures spread to the amygdala and upon amygdala stimulation. J Neurosci (2015) 35(28):10281–9. 10.1523/JNEUROSCI.0888-15.2015 26180203 PMC4502266

[70] Harper RM Bandler R Spriggs D Alger JR. Lateralized and widespread brain activation during transient blood pressure elevation revealed by magnetic resonance imaging. J Comp Neurol (2000) 417(2):195–204. 10.1002/(SICI)1096-9861(20000207)417:2<195::AID-CNE5>3.0.CO;2-V 10660897

[71] Resstel LBM Corrêa FMA Involvement of the medial prefrontal cortex in central cardiovascular modulation in the rat. Auton Neurosci (2006) 126:130–8. 10.1016/j.autneu.2006.02.022 16603420

[72] Guyenet PG The sympathetic control of blood pressure. Nat Rev Neurosci (2006) 7(5):335 10.1038/nrn1902 16760914

[73] Ziegler G Dahnke R Yeragani VK Bär KJ. The relation of ventromedial prefrontal cortex activity and heart rate fluctuations at rest. Eur J Neurosci (2009) 30(11):2205–10. 10.1111/j.1460-9568.2009.07008.x 20128855

[74] Wong SW Massé N Kimmerly DS Menon RS Shoemaker JK. Ventral medial prefrontal cortex and cardiovagal control in conscious humans. Neuroimage (2007) 35(2):698–708. 10.1016/j.neuroimage.2006.12.027 17291781

[75] Bär KJ Herbsleb M Schumann A de la Cruz F Gabriel HW Wagner G. Hippocampal-brainstem connectivity associated with vagal modulation after an intense exercise intervention in healthy men. Front Neurosci (2016) 10:145. 10.3389/fnins.2016.00145 27092046 PMC4823309

[76] Oppenheimer SM Gelb A Girvin JP Hachinski VC. Cardiovascular effects of human insular cortex stimulation. Neurology (1992) 42(9):1727–1727. 10.1212/WNL.42.9.1727 1513461

[77] Henderson LA Macey PM Macey KE Frysinger RC Woo MA Harper RK Brain responses associated with the Valsalva maneuver revealed by functional magnetic resonance imaging. J Neurophysiol (2002) 88(6):3477–86. 10.1152/jn.00107.2002 12466462

[78] Oppenheimer SM Kedem G Martin WM. Left-insular cortex lesions perturb cardiac autonomic tone in humans. Clin Auton Res (1996) 6(3):131–40. 10.1007/BF02281899 8832121

[79] Macey PM Wu P Kumar R Ogren JA Richardson HL Woo MA Differential responses of the insular cortex gyri to autonomic challenges. Auton Neurosci (2012) 168(1):72–81. 10.1016/j.autneu.2012.01.009 22342370 PMC4077282

[80] Chase MH Clemente CD Central neural components of the autonomic nervous system. J Am Soc Anesthesiol (1968) 29(4):625–33. 10.1097/00000542-196807000-00003 4874150

[81] Wilson CL Isokawa M Babb TL Crandall PH Levesque MF Engel J Functional connections in the human temporal lobe. Exp Brain Res (1991) 85(1):174–87. 10.1007/BF00229999 1884756

[82] Lacuey N Zonjy B Kahriman ES Kaffashi F Miller J Lüders HO Functional connectivity between right and left mesial temporal structures. Brain Struct Funct (2015) 220(5):2617–23. 10.1007/s00429-014-0810-0 24908158

[83] Shammah-Lagnado SJ Alheid GF Heimer L. Efferent connections of the caudal part of the globus pallidus in the rat. JComp Neurol (1996) 376(3):489–507. 10.1002/(SICI)1096-9861(19961216)376:3<489::AID-CNE10>3.3.CO;2-W 8956113

[84] Goetz CG Lutge W Tanner CM. Autonomic dysfunction in Parkinson’s disease. Neurology (1986) 36(1):73–73. 10.1212/WNL.36.1.73 3941786

[85] Goldstein DS Holmes C Li ST Bruce S Metman LV Cannon RO. Cardiac sympathetic denervation in Parkinson disease. Ann Intern Med (2000) 133(5):338–47. 10.7326/0003-4819-133-5-200009050-00009 10979878

[86] Cohen MX Schoene-Bake JC Elger CE Weber B. Connectivity-based segregation of the human striatum predicts personality characteristics. Nat Neurosci (2009) 12(1):32. 10.1038/nn.2228 19029888

[87] Robinson JL Laird AR Glahn DC Blangero J Sanghera MK Pessoa L The functional connectivity of the human caudate: an application of meta-analytic connectivity modeling with behavioral filtering. Neuroimage (2012) 60(1):117–29. 10.1016/j.neuroimage.2011.12.010 22197743 PMC3288226

[88] Stoffers D Altena E van der Werf YD Sanz-Arigita EJ Voorn TA Astill RG The caudate: a key node in the neuronal network imbalance of insomnia? Brain (2014) 137(2):610–20. 10.1093/brain/awt329 24285642 PMC3914473

[89] Borsook D Upadhyay J Chudler EH Becerra L A key role of the basal ganglia in pain and analgesia-insights gained through human functional imaging. Mol Pain (2010) 6(1):27 10.1186/1744-8069-6-27 20465845 PMC2883978

[90] Grahn JA Parkinson JA Owen AM. The cognitive functions of the caudate nucleus. Prog Neurobiol (2008) 86(3):141–55. 10.1016/j.pneurobio.2008.09.004 18824075

[91] Woo MA Kumar R Macey PM Fonarow GC Harper RM. Brain injury in autonomic, emotional, and cognitive regulatory areas in patients with heart failure. J Card Fail (2009) 15(3):214–23. 10.1016/j.cardfail.2008.10.020 19327623 PMC2730774

[92] Kumar R Nguyen HD Ogren JA Macey PM Thompson PM Fonarow GC Global and regional putamen volume loss in patients with heart failure. Eur J Heart Fail (2011) 13(6):651–5. 10.1093/eurjhf/hfr012 21393297 PMC3101866

[93] Harper RM Kumar R Ogren JA Macey PM. Sleep-disordered breathing: effects on brain structure and function. Respir Physiol Neurobiol (2013) 188(3):383–91. 10.1016/j.resp.2013.04.021 23643610 PMC3778068

[94] Harper RM Kumar R Macey PM Woo MA Ogren JA. Affective brain areas and sleep-disordered breathing. Prog Brain Res (2014) 209:275–93. 10.1016/B978-0-444-63274-6.00014-X 24746053 PMC4060533

[95] Park B Palomares JA Woo MA Kang DW Macey PM Yan-Go FL Aberrant Insular Functional Network integrity in patients with obstructive sleep apnea. Sleep (2016) 39(5):989–1000. 10.5665/sleep.5738 26943471 PMC4835320

[96] Kumar R Farahvar S Ogren JA Macey PM Thompson PM Woo MA Brain putamen volume changes in newly-diagnosed patients with obstructive sleep apnea. Neuroimage Clin (2014) 4:383–91. 10.1016/j.nicl.2014.01.009 24567910 PMC3930100

[97] Ogren JA Macey PM Kumar R Woo MA Harper RM. Central autonomic regulation in congenital central hypoventilation syndrome. Neuroscience (2010) 167(4):1249–56. 10.1016/j.neuroscience.2010.02.078 20211704 PMC3003708

[98] Kumar R Macey PM Woo MA Alger JR Keens TG Harper RM. Neuroanatomic deficits in congenital central hypoventilation syndrome. JComp Neurol (2005) 487(4):361–71. 10.1002/cne.20565 15906312

[99] Kumar R Macey PM Woo MA Harper RM. Rostral brain axonal injury in congenital central hypoventilation syndrome. J Neurosci Res (2010) 88(10):2146–54. 10.1002/jnr.22385 20209631

[100] Macey PM Moiyadi AS Kumar R Woo MA Harper RM Decreased cortical thickness in central hypoventilation syndrome. Cereb Cortex (2011) 22(8):1728–37. 10.1093/cercor/bhr235 21965438 PMC3500857

[101] Ansakorpi H Korpelainen JT Huikuri HV Tolonen U Myllylä VV Isojärvi JIT. Heart rate dynamics in refractory and well controlled temporal lobe epilepsy. J Neurol Neurosurg Psychiatry (2002) 72(1):26–30. 10.1136/jnnp.72.1.26 11784820 PMC1737701

[102] Stein PK Bosner MS Kleiger RE Conger BM Heart Rate Variability: A measure of cardiac autonomic tone. Am Heart J (1993) 127:1376–81. 10.1016/0002-8703(94)90059-0 8172068

[103] Strange BA Witter MP Lein ES Moser EI. Functional organization of the hippocampal longitudinal axis. Nat Rev Neurosci (2014) 15(10):655–69. 10.1038/nrn3785 25234264

[104] Behrens TEJ Johansen-Berg H Woolrich MW Smith SM Wheeler-Kingshott CAM Boulby PA Non-invasive mapping of connections between human thalamus and cortex using diffusion imaging. Nat Neurosci (2003) 6(7):750–7. 10.1038/nn1075 12808459

[105] Ogren JA Macey PM Kumar R Fonarow GC Hamilton MA Harper RM Impaired cerebellar and limbic responses to the valsalva maneuver in heart failure. Cerebellum (2012) 11(4):931–8. 10.1007/s12311-012-0361-y 22370874

[106] Ridley BGY Rousseau C Wirsich J Le Troter A Soulier E Confort-Gouny S Nodal approach reveals differential impact of lateralized focal epilepsies on hub reorganization. Neuroimage (2015) 118:39–48. 10.1016/j.neuroimage.2015.05.096 26070261

[107] Cullen KR Westlund MK Klimes-Dougan B Mueller BA Houri A Eberly LE Abnormal amygdala resting-state functional connectivity in adolescent depression. JAMA Psychiatry (2014) 71(10):1138–47. 10.1001/jamapsychiatry.2014.1087 25133665 PMC4378862

[108] Sawaya H Johnson K Schmidt M Arana A Chahine G Atoui M Resting-state functional connectivity of antero-medial prefrontal cortex sub-regions in major depression and relationship to emotional intelligence. Int J Neuropsychopharmacol (2015) 18:yu112. 10.1093/ijnp/pyu112 25744282 PMC4438550

[109] Thapar A Kerr M Harold G. Stress, anxiety, depression, and epilepsy: investigating the relationship between psychological factors and seizures. Epilepsy Behav (2009) 14(1):134–40. 10.1016/j.yebeh.2008.09.004 18824131

