# Supplementary material for: Dysfunctional Brain Networking among Autonomic Regulatory Structures in Temporal Lobe Epilepsy Patients at High Risk of Sudden Unexpected Death in Epilepsy
Source: Front Neurol. 2017 Oct 16;8:544. doi: 10.3389/fneur.2017.00544 (PMC5650686; doi:10.3389/fneur.2017.00544)
Supplement: Supplementary file 1 [file Table_1.DOCX]

**Methods**

**Subjects and risk stratification**

| Patient | Risk | No. of AEDs | Medication (type, dose [mg]) |
| --- | --- | --- | --- |
| 1 | High | 1 | LEV, 1000 BD |
| 2 | High | 2 | CBZ CR, 600 BD; LEV, 1500 BD |
| 3 | High | 3 | LEV, 1000/2000; VAL, 1000/1500; ZNS, 75 BD |
| 4 | High | 2 | LEV, 1750 BD; PHT, 200/250 |
| 5 | High | 3 | CBZ, 400 BD; TPM, 100 BD; PGB, 25 OD |
| 6 | High | 1 | TEG R, 200/400 |
| 7 | High | 2 | PHT, 300 on; LEV, 1500 BD |
| 8 | High | 3 | LEV, 1500 BD; TEG retard, 400 BD; LAC, 150 BD |
| 9 | High | 3 | LEV, 1500 BD; OXC, 600 BD; TPM, 100 BD |
| 10 | High | 3 | PER, 4 ON; TPM, 50 BD; LAC, 200 BD |
| 11 | High | 3 | CBZ CR, 600/800; ACTZ 500 BD; PB, 30 OD |
| 12 | High | 2 | LEV, 1500 BD; CBMZ, 600/800; CLBZ, 20 ON |
| 13 | High | 3 | OXC, 600 BD; CLB 20 ON; GBP, 100 TDS |
| 14 | High | 2 | LTG, 500 BD; LAC, 200 BD |
| 15 | Low | 3 | LEV, 1250 BD; LTG, 75 BD; TEG SR, 600 BD |
| 16 | Low | 2 | TPM, 175 BD; LTG 250 BD |
| 17 | Low | 2 | LEV, 200 BD; CBZ, 600 BD |
| 18 | Low | 2 | ZNS, 200 BD; VAL CR, 500 BD |
| 19 | Low | 3 | CBZ PR, 400 BD; LEV, 1500/1000; ZNS, 75 BD |
| 20 | Low | 2 | LEV, 2000 BD; LTG, 175 BD |
| 21 | Low | 3 | TEG PR, 600 BD; LTG, 250 BD; ZNS, 250 BD |
| 22 | Low | 3 | CBZ, 400/600; LEV, 1000mg BD; LTG, 150 BD |
| 23 | Low | 2 | LEV, 1000 BD; TPM, 300 BD |
| 24 | Low | 1 | TEG PR 800, 1000 |
| 25 | Low | 3 | PHT, 100 BD; LAC, 100 BD; LEV, 500 BD |
| 26 | Low | 4 | TEG PR, 800 / 600; EP-C, 1000 BD; LEV, 1500 BD; CLB, 20 ON |
| 27 | Low | 2 | LTG, 200 BD; LEV, 250 BD |
| 28 | Low | 1 | EP, 1000 BD |
| 29 | Low | 1 | TPM, 100 BD |
| 30 | Low | 1 | CBZ, 200 TDS |
| 31 | Low | 2 | LEV, 1000BD; OXC 900 BD |
| 32 | Low | 2 | OXC, 450/600; LEV, 1250 BD |

**Supplementary medication table:** Anti-epileptic drug (AED) type, number and dosages for high- and low-risk SUDEP patients.

**AED table legend:** mg = milligrams, OD = once daily, ON = every night, BD = bis die (twice a day), TDS = three times daily, CR = controlled release, PR = prolonged release, SR = Sustained release, ACTZ = Acetazolamide, CBZ = Carbamazepine, CLB = Clobazam, EP = Epilim, EP-C = Epilim Chrono, GBP = Gabapentin, LAC = Lacosamide, LTG = Lamotrigine, LEV = Levetiracetam, OXC = Oxcarbazepine, PER = Perampanel, PB = Phenobarbital, PHT = Phenytoin, PGB = Pregabalin, TEG = Tegretol, TPM = Topiramate, VAL = Sodium Valproate, ZNS = Zonisamide.
