# Supplementary material for: Dysfunctional Brain Networking among Autonomic Regulatory Structures in Temporal Lobe Epilepsy Patients at High Risk of Sudden Unexpected Death in Epilepsy
Source: Front Neurol. 2017 Oct 16;8:544. doi: 10.3389/fneur.2017.00544 (PMC5650686; doi:10.3389/fneur.2017.00544)
Supplement: Supplementary file 2 [file Data_Sheet_1.DOCX]

**Methods**

**Pre-processing (regression of hippocampal gray matter volume)**

As well as using presence of hippocampal sclerosis as a covariate, we also performed analysis with hippocampal volume of epileptogenic hemisphere regressed out. To do this, hippocampal volumes were calculated for each patient using the Harvard-Oxford sub-cortical atlas hippocampal (left and right where applicable) masks. For each patient, the respective ROI mask (depending on lateralisation of epilepsy) was used to extract the amount gray matter volume within the mask using the normalised gray matter segmentations created in pre-processing via DARTEL. This yielded a value representing the total amount of gray matter volume within the mask. This was then used as a covariate in statistical analysis within the NBS formalism, replacing the binary ‘presence or non-presence of hippocampal sclerosis’ covariate.

**Results**

Two significant subnetworks emerged following regression of hippocampal gray matter volume (instead of ‘presence of hippocampal sclerosis’). The high-risk < low-risk contrast revealed a significantly reduced subnetwork of 11 nodes and 14 edges (*t* = 2.5, *p* = .035), while the high-risk > low-risk contrast showed a significantly enhanced subnetwork comprising 14 nodes and 27 edges (*t* = 2.5, *p* = .028).


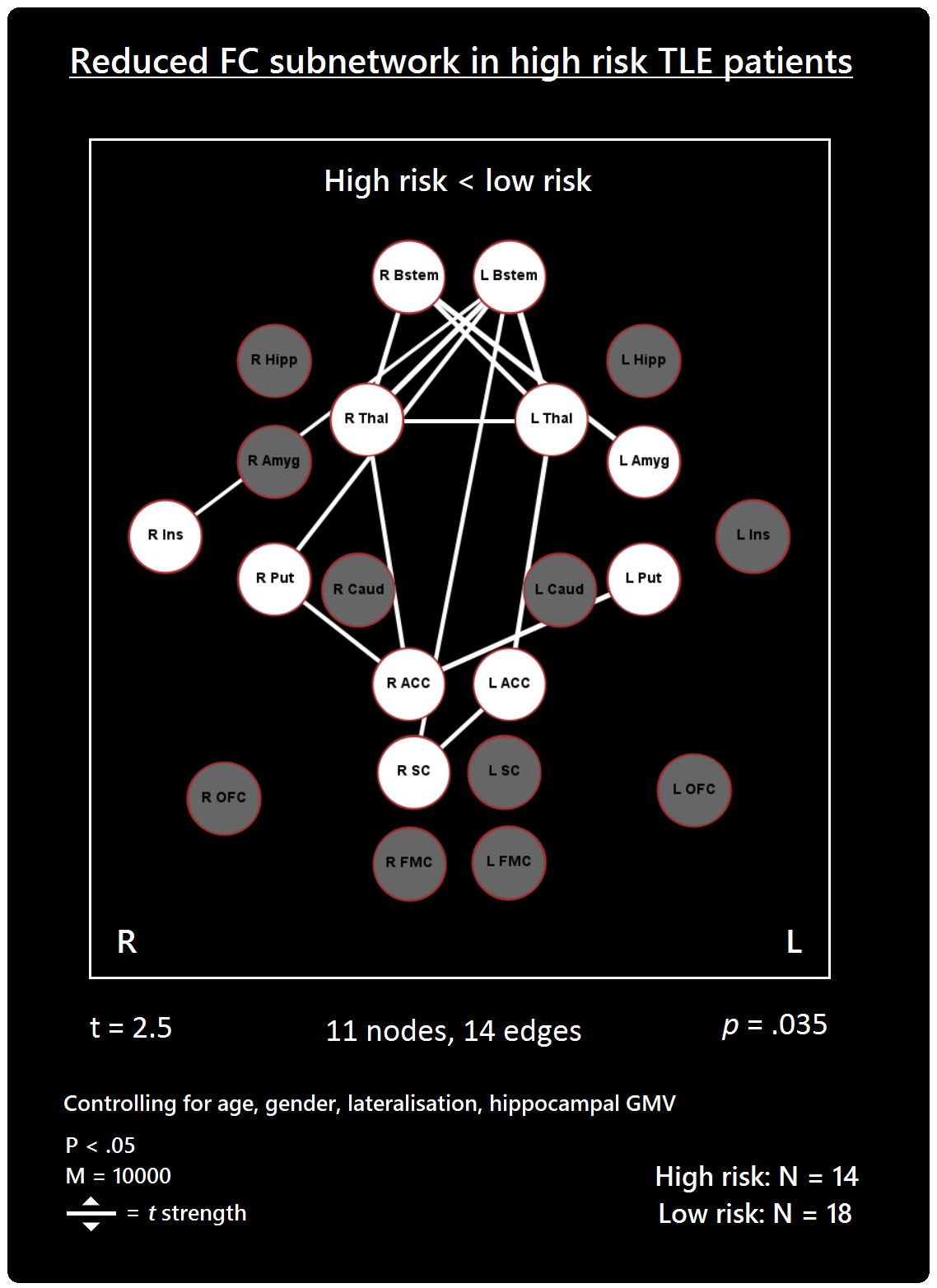


**Supplementary Figure 1:** Reduced FC subnetwork with hippocampal gray matter volume of epileptogenic hemisphere, not presence of HS, regressed out. Subnetwork involves the bilateral brainstem (Bstem), bilateral thalamus (Thal), bilateral putamen (Put), bilateral ACC, left amygdala (Amyg), right subcallosal cortex (SC) and right Insula (Ins). L = Left, R = Right; t = t-statistic threshold; M = number of permutations; P value was set at < 0.05, FWER corrected. Nodes in white are those which were involved in the significant subnetwork. Red node outline represents search for reduced connectivity (high<low). Visualisation using Gephi (https://gephi.org/).


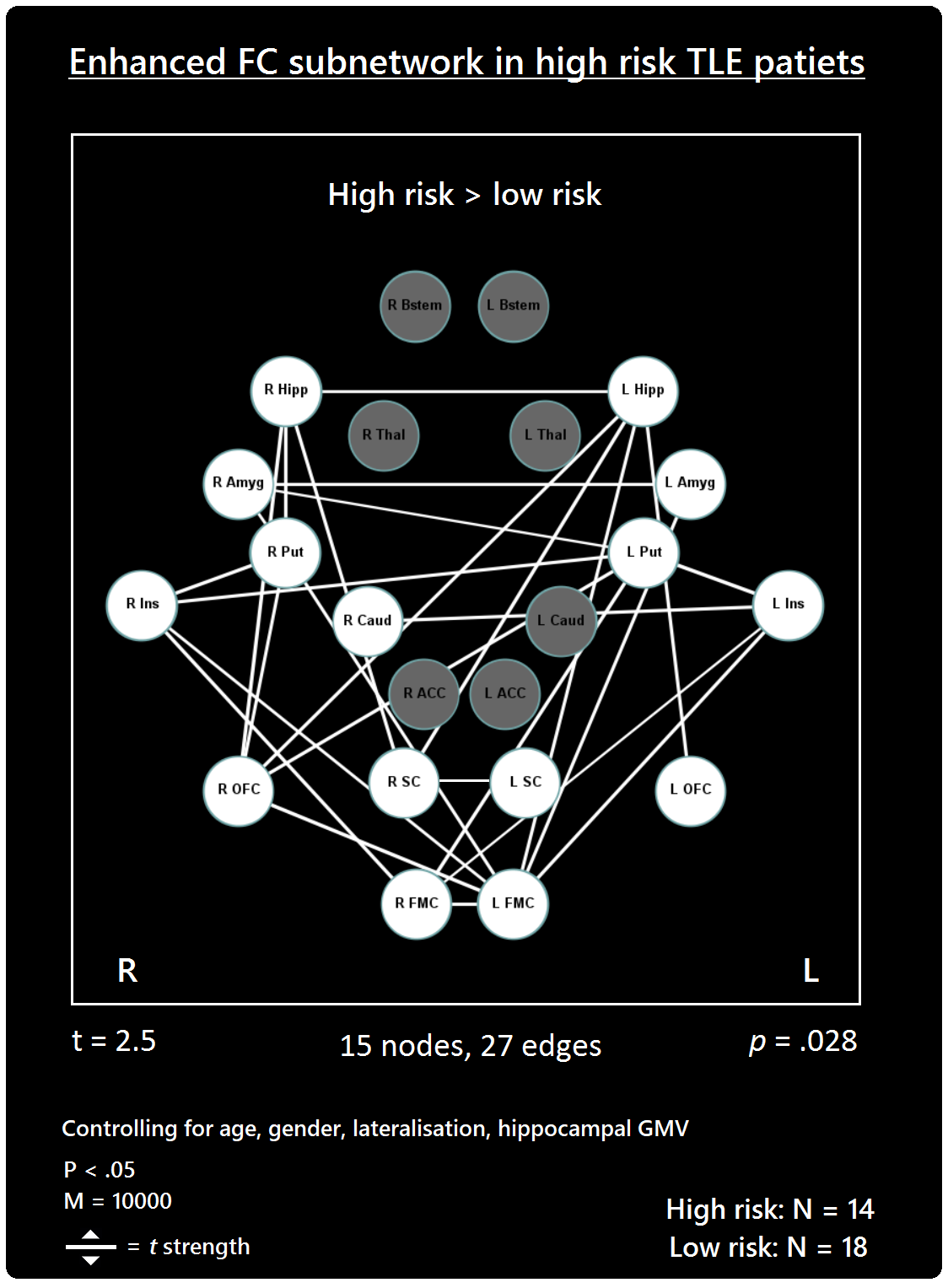


**Supplementary Figure 2:** Subnetwork of enhanced FC in high-risk TLE patients when compared with low-risk TLE patients, with hippocampal gray matter volume of epileptogenic hemisphere regressed out. Regions include: bilateral amygdala (L Amyg, R Amyg), right caudate (R Caud), bilateral frontal medial cortex (L FMC, R FMC), bilateral hippocampus (L Hipp, R Hipp), bilateral insula (L Ins, R Ins), bilateral orbitofrontal cortex (L OFC, R OFC), bilateral putamen (L Put, R Put), and left and right subcallosal cortex (L SC, R SC). L = left, R = Right; t = t-statistic threshold; M = number of permutations; P value was set at < 0.05, FWER corrected. White nodes represent ROIs involving significant connections. Blue node outline represents search for increased connectivity (high>low). Visualisation using Gephi (https://gephi.org/).
